# Potential antivirulence and antibiofilm activities of sub-MIC of oxacillin against MDR *S. aureus* isolates: an in-vitro and in-vivo study

**DOI:** 10.1186/s12866-024-03429-8

**Published:** 2024-08-09

**Authors:** Amira Omar, Tarek E. El-Banna, Fatma I. Sonbol, Maisra M. El-Bouseary

**Affiliations:** Department of Microbiology and Immunology, Faculty of Pharmacy, Tanta University, Tanta, Egypt

**Keywords:** *S. aureus*, Virulence factors, Biofilm, Oxacillin, Sub-MIC

## Abstract

**Background:**

Multi-drug resistant *Staphylococcus aureus* is one of the most common causes of nosocomial and community-acquired infections, with high morbidity and mortality. Treatment of such infections is particularly problematic; hence, it is complicated by antibiotic resistance, and there is currently no reliable vaccine. Furthermore, it is well known that *S. aureus* produces an exceptionally large number of virulence factors that worsen infection. Consequently, the urgent need for anti-virulent agents that inhibit biofilm formation and virulence factors has gained momentum. Therefore, we focused our attention on an already-approved antibiotic and explored whether changing the dosage would still result in the intended anti-virulence effect.

**Methods:**

In the present study, we determined the antibiotic resistance patterns and the MICs of oxacillin against 70 MDR *S. aureus* isolates. We also investigated the effect of sub-MICs of oxacillin (at 1/4 and 1/8 MICs) on biofilm formation using the crystal violet assay, the phenol-sulphuric acid method, and confocal laser scanning microscopy (CLSM). We examined the effect of sub-MICs on virulence factors and bacterial morphology using quantitative reverse transcription polymerase chain reaction (qRT-PCR) and electron microscopy, respectively. Moreover, we studied the effect of sub-MICs of oxacillin (OX) in-vivo using a wound infection model.

**Results:**

Oxacillin at 1/2 MIC showed a significant decrease in bacterial viability, while 1/4 and 1/8 MICs had negligible effects on treated bacterial isolates. Treatment of MDR isolates with 1/4 or 1/8 MICs of oxacillin significantly reduced biofilm formation (64% and 40%, respectively). The treated MDR *S. aureus* with sub-MICs of OX exhibited a dramatic reduction in several virulence factors, including protease, hemolysin, coagulase, and toxic shock syndrome toxin-1 (TSST-1) production. The sub-MICs of OX significantly decreased (*P* < 0.05*)* the gene expression of biofilm and virulence-associated genes such as *agrA*, *icaA*, *coa*, *and tst.* Furthermore, oxacillin at sub-MICs dramatically accelerated wound healing, according to the recorded scoring of histological parameters.

**Conclusion:**

The treatment of MDR *S. aureus* with sub-MICs of oxacillin can help in combating the bacterial resistance and may be considered a promising approach to attenuating the severity of *S. aureus* infections due to the unique anti-biofilm and anti-virulence activities.

**Supplementary Information:**

The online version contains supplementary material available at 10.1186/s12866-024-03429-8.

## Background

*Staphylococcus aureus* is one of the most prevalent bacteria that cause serious illness. Pneumonia, cardiovascular disease, prosthetic joints, surgical sites, nosocomial bacteremia, and late-onset septicemia in infants are frequently caused by *S. aureus* [1–3]. Several factors complicate *S. aureus* infections and affect their pathogenicity, like antibiotic resistance and the capacity of biofilm and virulence factor production [4].

*S. aureus* infection severity is determined by the virulence factors produced, such as hemolytic toxins (a, b, c, and d), protease, coagulase, and toxic shock syndrome toxin-1 (TSST-1), and the production of these factors is regulated by several regulatory loci, including the accessory gene regulator (*agr)* and staphylococcal accessory regulator (*sarA*) genes [5–7]. Moreover, *S. aureus* infections, particularly those associated with biofilm formation, are resistant to several types of antibiotics [8, 9]. The biofilm shields bacteria from numerous environmental stresses and prevents antibiotic penetration and interaction with bacterial cells [10]. Therefore, a challenging problem in clinical practice is that *S. aureus* is becoming increasingly resistant to different antibiotic treatments, underscoring the need for alternate therapeutic approaches [5, 11, 12]. Consequently, targeting the *S. aureus* biofilm and virulence factor production is one of the best approaches to preventing the occurrence of resistance.

The approach referred to as “anti-virulence therapy” is an innovative breakthrough in the battle against infections caused by bacteria. Instead of killing the microbes, its goal is to reduce their virulence-related characteristics [12]. Although virulence features such as biofilms, enzymes, and toxins may be expressed differently in various species of bacteria, their regulation processes are almost comparable. Interfering with the regulatory mechanisms of virulence expression is a key goal of antivirulence medications [13]. The lack of clinical trials and non-approval for many antivirulence agents that have been studied to date in vitro and/or in laboratory animals has hampered the development of antivirulence therapeutics [14]. Anti-virulent drugs that safely prevent the production of biofilms and virulence factors are therefore desperately needed. Finding repurposing candidates with antivirulence characteristics is extremely crucial. This strategy certainly has the potential to advance research on antivirulence medications, but there are still several significant problems that need to be resolved before antivirulence drugs can compete with traditional antibiotics [15]. Therefore, we aimed to reconsider the use of oxacillin with modifications in its concentration to overcome the increase in microbial resistance through the assessment of its impact on bacterial biofilm and virulence factor production.

## Materials and methods

### Bacterial isolation and identification

The clinical samples (sputum (*n* = 78), urine (*n* = 31), wound (*n* = 7), and blood (*n* = 4)) were obtained from patients admitted to Tanta University Hospitals, Tanta, Egypt, and cultivated on nutrient agar after being grown in nutrient broth (Oxoid, UK). The recovered bacterial colonies were subjected to traditional identification procedures, such as Gram staining, cultivation on mannitol salt agar plates (Oxoid, UK), and performing common biochemical methods for identification like coagulase, catalase, and the methyl red test [16].

### Antibiotic susceptibility testing (AST)

The antibiotic susceptibility testing was carried out for the following antibiotic discs (Oxoid, UK); penicillin (P; 10 µg), oxacillin (OX; 1 µg), cefoxitin (FOX; 30 µg), azithromycin (AZM; 15 µg), chloramphenicol (C; 30 µg), trimethoprim-sulfamethoxazole (SXT; 1.25/23.75 µg), linezolid (LZ; 30 µg), levofloxacin (LEV; 5 µg), tetracycline (T; 30 µg), clindamycin (CD; 2 µg), rifampicin (RA; 5 µg) and vancomycin (V; 30 µg), ciprofloxacin (CIP; 5 µg), gentamicin (GN; 10 µg), amikacin (AK; 30 µg). Multiple drug-resistant (MDR) isolates are those that are resistant to one antimicrobial drug in at least three or more antimicrobial categories [17].

### Minimum inhibitory concentrations (MIC) of recovered MDR isolates

The agar dilution assay was performed according to CLSI standards to determine the MIC values of oxacillin against *S. aureus* isolates. Briefly, a basal medium called Muller-Hinton agar (MHA) (Oxoid, UK) was employed. In a water bath, sterile Mueller-Hinton agar (MHA) was allowed to acclimate to a temperature of roughly 50 °C. Oxacillin stock solution was produced using a membrane filter. The molten MHA was poured into Petri dishes after the chosen antimicrobial agent was added at escalating concentrations (multiple of two, i.e., 0.25, 0.5, 1, 2, 4,. 1024 µg/ml). After solidification of the agar, the bacteria suspension (10^4^ CFU/ml) was inoculated on the agar plate surface with a specific microbial inoculum and incubated overnight at 37 °C [18, 19].

### Screening of biofilm production by the MDR isolates

Using 96-well flat-bottom plates to investigate biofilm formation briefly, we performed bacterial suspensions in TSB (Oxoid, USA) from overnight cultures. Injecting the wells with 100 µl of bacterial suspension (10^6^ CFU/ml), the wells were incubated for 24 h at 37 °C. Then, fix the formed biofilm using 100% methanol for 20 min and dry it. After that, using phosphate-buffered saline (PBS) to gently clean the plates, they were dyed with 100 µl of 0.1% crystal violet (CV) (BDH, UK) for 30 min. After washing, the extra CV was eliminated, and biofilm was assessed by using a microtiter reader (Sunrise TM, TECAN, Switzerland), which measured the OD value at 595 nm after CV was dissolved in 33% (v/v) glacial acetic acid (Prolabo, France) [20, 21]. The uninoculated medium served as a negative control. The assay was carried out in triplicate.

As previously described by Stepanovic et al. [22], the cutoff value (ODc) was defined as three standard deviation units above the mean absorbance of the negative control. The isolates were subsequently categorized as follows: non-biofilm-forming (OD ≤ ODc), weak biofilm-forming (ODc ≥ lOD ≤ 2ODc), moderate biofilm-forming (2ODc ≥ OD ≤ 4ODc), and strong biofilm-forming isolates (OD ≥ 4ODc).

### Screening for virulence factor expression by the MDR isolates

About 25 g of non-fat dry milk was reconstituted with 250 ml of distilled water. The mixture was stirred thoroughly and autoclaved at 121 °C for 15 min. A volume of 250 ml of 1.5% LB agar (Oxoid, UK) solution was sterilized. After that, skim milk and LB agar solutions were held in a water bath at 50 °C, and then the skim milk was poured into the agar bottle and mixed thoroughly. The mixture was then dispensed into sterile plates, and then the tested isolates were cultivated. We looked at the plates to see if clear zones had developed around the inoculated bacteria after incubation for 48 h at 28 °C. The hemolytic activity was also investigated by investigating the lysis zones around the inoculated bacteria after 48 h at 28 °C, and this was performed on the blood agar plates prepared according to the following steps: To the sterile LB agar (Oxoid, UK), which has been melted and cooled to 45–50 °C, sterile defibrinated blood (4% v/v) that has been warmed to room temperature was added, mixed thoroughly by swirling the flask, and the mixture was dispensed into sterile plates on which *S. aureus* isolates were cultivated. TSST-1 was detected using a screening PCR technique [23, 24].

### The growth curve of MDR isolates before and after oxacillin treatment

The isolates (*n* = 13) that produce all virulence factors under investigation were cultivated in LB (Oxoid, UK) broth both with and without 1/2, 1/4, and 1/8 MICs of OX at 37 °C with an OD600 of 0.3. at 30-min intervals. Samples were taken and the absorbance was measured at 600 nm; 3 ml of each culture were taken at zero time, 30, 60, 90, 120, 150, 180, 210, 240, 270, 300, 330, 360, and 420 min [25].

### Testing the effect of oxacillin at sub-MIC on *S. aureus* biofilm formation

#### Crystal violet microtiter plate assay

The impact of OX on *S. aureus* isolates’ biofilm was investigated, as prescribed by Saeloh et al. [26]. Briefly, making use of a 96-well microtiter plate, the isolates (10^6^ CFU/ml) were cultured in TSB (Oxoid, UK) for 24 h at 37 °C with and without 1/8 MIC and 1/4 MIC of OX. Fixing the formed biofilm was performed using 100% methanol for 20 min and then drying. The biofilm was stained with 200 µl of 0.1% crystal violet (BDH, UK) for 15 min. The plate was then dried after rinsing with water. Dissolving stained biofilms with 200 µl glacial acetic acid (Prolabo, France) with a concentration of 33% (v/v) and an OD of 595 nm was evaluated using a microplate reader (Sunrise TM, TECAN, Switzerland).

#### Phenol-sulfuric acid method

The impact of oxacillin on *S. aureus* biofilm matrix production (exopolysaccharide) EPS was investigated. The isolates were cultured in LB broth (Oxoid, UK) with and without 1/8 and 1/4 MIC OX and cultivated for 24 h at 37 °C. After that, bacterial suspensions (10^6^ CFU/ml) were centrifuged for 10 min at 8000 g, and the pellets were centrifuged once more after being re-suspended in PBS. A similar volume of ethyl alcohol (Sigma, USA) was added to the supernatant after centrifuging. Finally, the EPS solution (1 ml), cold 5% phenol (Sigma, USA) (1 ml), and concentrated sulfuric acid (Sigma, USA) (5 ml) were properly blended. Following OX treatment, the OD was measured at 490 nm, and from this value, the percentages of EPS reduction were estimated [27].

#### Testing biofilm thickness and bacterial viability using confocal laser scanning microscopy (CLSM)

The impact of OX on *S. aureus* biofilm was detected using CLSM. The 8-well slide (ibid., Martinsried, Germany) was used to cultivate the selected bacterial suspension (10^6^ CFU/ml) before and after OX treatment at 1/8 and 1/4 MICs. After being incubated for 18 h at 37 °C with the use of PBS, the microplates were washed twice and then dyed with 5 µl of propidium iodide (PI) (Sigma, USA) and acridine orange (Sigma, USA) (AO), which give red fluorescence due to the staining of dead cells and green fluorescence due to the staining of live cells, respectively, for 15 min in the dark. Finally, we investigated the biofilm using CLSM (DMi8, Leica Microsystem, USA) [28].

### Testing of sub-MIC of oxacillin on MDR virulence factors

#### Protease production

Isolates screened on skimmed milk for protease production were cultivated in LB broth (Oxoid, UK) with and without OX for 24 h at 37 °C. Then the broth was filtered through a filter (0.45 μm) after centrifugation. The skim milk agar plates’ wells were filled with filtrated supernatants (100 µl). After 24 h at 37 °C, the size of the lysis zones that had developed around the wells was measured. Also, we performed a protease assay using casein (Sigma, USA) as a substrate by making a bacterial suspension (10^6^ CFU/ml) in LB broth with and without OX and incubating for 24 h, taking 1 ml of the supernatant after centrifugation and filtration, then adding 1 ml of 0.05 M phosphate buffer and 0.1 M NaOH containing 2% casein and incubating at 37 °C for 10 min. The stopping of the reaction was achieved by adding 2 ml of 0.4 M TCA (Sigma, USA) and incubating at 37 °C for 30 min, followed by centrifugation at 5000 rpm for 15 min. Finally, we mixed 3 ml of filtrate, 5 ml of Na_2_CO_3_, and 2 ml of Folin (Sigma, USA), and measured the absorbance at 660 nm [29].

#### Hemolysin production

The impact of OX on hemolysin production was determined using qualitative and quantitative methods. In the qualitative method, bacterial overnight culture (20 µl) was injected into 180 µl LB broth (10^6^ CFU/ml), and then the broth was incubated both with and without OX for 18 h at 37 °C. The samples were then streaked on human blood agar for 24 h at 37 °C. Then, the clear zone around the inoculated bacteria was visualized. Concerning the quantitative method, *S. aureus* isolates were cultivated in LB broth with and without OX. We mixed 600 ml of centrifuged and filtrated supernatant with 600 ml of a 2% suspension of red blood cells (RBCs) and incubated for 2 h at 37 °C, then centrifuged at 10,000 rpm for 8 min at 4 °C, measuring OD at 540 nm to determine hemoglobin release [5, 30].

#### Coagulase production

The supernatants were double-fold diluted serially in 96-well, round-bottom microtiter plates using brain heart infusion (BHI) broth (Oxoid, UK). All the wells received equal amounts of a citrated plasma solution (20% v/v) before being combined. Incubating the plates at 37 °C and observing the plates after 4 h of incubation. The titer, which is the reciprocal of the greatest dilution showing plasma coagulation, was recorded. By combining citrated rabbit plasma with BHI medium, negative controls were employed. The experiment was repeated three times [31–33].

### Morphological changes induced by sub-MIC of OX

#### Scanning electron microscope (SEM)

Bacteria suspension (10^6^ CFU/ml) was cultivated for 24 h on polystyrene plates with 24 wells (Thermo Scientific, USA) with and without OX at 1/4 MIC, then the cultural media was removed, and the plates were washed. For 20 min at 4 °C, ice-cold 3% glutaraldehyde (Sigma, USA) was used to fix the entire well. An inspect S microscope operating at 15 or 20 KV was used to examine the wells after they had been dehydrated using ethanol (Sigma, USA), dried using air-dried sputter-coated gold, and then examined using SEM (S-34,002 N SEM, Hitachi^®^, Tokyo, Japan) [34].

#### Transmission electron microscope (TEM)

The bacterial suspension (10^6^ CFU/ml) was both with and without a 1/8 MIC of OX during incubation for 24 h at 37 °C, and the bacterial cell morphology was examined on 100-mesh Cu grids supported by the carbon-coated formvar film. Cells were applied and then allowed to dry following a 20 s negative stain with 1% phosphotungstic acid (Sigma, USA). The cells were studied using TEM (JEM-1011, Gatan^®^, USA) [34].

Morphological modifications brought on by 1/8 MIC of OX were determined as follows: the bacterial suspension (10^6^ CFU/ml) was both with and without a 1/8 MIC of OX during incubation at 37 °C for 24 h. The sample was washed three times with PBS. Using 2.5% glutaraldehyde in PBS (pH 7.4), the pellets were fixed. Then, it was rinsed and postfixed for 2 h in 1% OsO4 in PBS buffer (pH 7.4). The sample was dehydrated using ethanol (Sigma, USA) and implanted in epoxy resin Epon 812 (Sigma, USA), then ultrathin sections were cut and stained with uranyl acetate and lead citrate (Sigma, USA), then observed using TEM (TEM-2100, JEOL^®^, Tokyo, Japan) [35].

#### Investigating the mechanism of action of sub-MIC of oxacillin on MDR biofilm and virulence factors using qRT-PCR

In LB broth, *S. aureus* isolates were grown without and with OX. The isolate pellets obtained after centrifugation were processed using an RNA extraction kit as directed by the producer (Roche Diagnostic GmbH, Germany). Samples with a 260/280 nm ratio in the 1.8–2 range were used. The complementary DNA (cDNA) synthesis was performed as directed by the manufacturer’s procedures (ThermoFisher Scientific, Waltham, MA, USA). To evaluate the gene transcript levels using the oligonucleotides mentioned in Table 1., qRT-PCR was carried out using Rotor-Gene Q (Qiagen, USA). The 16 S rRNA served as a housekeeping gene.

**Table 1:** Sequence of primers used in qRT-PCR

Genes	Oligonucleotides	Reference
***16 S rRNA***	F: GCTGCCCTTTGTATTGTC R: AGATGTTGGGTTAAGTCCC	[36]
***agrA***	F: GCACATACACGCTTACAATTGTTG R: ACACTGAATTACTGCCACGTTTTAAT	[36]
***icaA***	F: GAGGTAAAGCAACGCACTC R: CCTGTAACCGCACCAAGTTT	[37]
***coa***	F: CGAGACCAAGATTCAACAAG R: AAAGAAAACCACTCACATCA	[24]
***tst***	F-CATCTACAAACGATAATATAAAGG R-CATTGTTATTTTCCAATAACCACCCG	[24]

### In-vivo study using wound infection model

### Animals

Thirty male Wistar albino rats were obtained from the animal house located at the faculty of veterinary medicine, Cairo University, Egypt. Animals weighed 120–150 g and were supplied with filtered water and standard pellets at 25 ± 2 °C and 12 h light/dark cycles. The in vivo methods and protocol were accredited by the Research Ethical Committee (Faculty of Pharmacy, Tanta University, Egypt) as aligned with the standard rules of handling and caring for laboratory animals. (TR/RE/8/23 p-0038).

### Experimental design and animal groups

Rats were randomly assigned into three groups (10 rats per group). Group I was the positive control group (received bacteria without treatment). Group II and Group III received bacteria and were treated with 1/4 and 1/8 MICs of OX, respectively. A wound infection model was performed, and rats were housed separately after wound-making to avoid fighting and cross-contamination. The back of each rat was cleaned with 10% povidone-iodine after they had their hair cut. Under the anesthesia of xylazine (5 mg/kg) and ketamine (40 mg/kg), biopsy punches were used to create two 10 mm-thick excisional lesions on the rat’s dorsal portion on each side of the spine. Each mouse served as a separate healing as one side received 20 µl of PBS (negative control) and the other side received bacteria (10 µl) with 16 µg/ml and 8 µg/ml of OX, which represent 1/4 and 1/8 MIC, respectively, with or without treatment. The infection of wounds was achieved by the addition of 10 µl of the microbial suspension (10^6^ CFU). PBS was used to prepare and dilute the bacterial suspension. 20 µl of vehicle (PBS) and oxacillin at 1/4 and 1/8 MIC were administered to the wounds after 30 min of bacterial inoculation. On days 0, 3, and 6, pictures of the wounds were taken using Image J software version 146 and measuring the size of the wound. The percentage of wound closure was calculated using the following equation:

$$\:\frac{\text{O}\text{r}\text{i}\text{g}\text{i}\text{n}\text{a}\text{l}\:\text{w}\text{o}\text{u}\text{n}\text{d}\:\text{s}\text{i}\text{z}\text{e}-\text{W}\text{o}\text{u}\text{n}\text{d}\:\text{s}\text{i}\text{z}\text{e}\:\text{a}\text{t}\:\text{t}\text{h}\text{e}\:\text{t}\text{i}\text{m}\text{e}\:\text{o}\text{f}\:\text{t}\text{a}\text{k}\text{i}\text{n}\text{g}\:\text{t}\text{h}\text{e}}{\text{I}\text{n}\text{i}\text{t}\text{i}\text{a}\text{l}\:\text{w}\text{o}\text{u}\text{n}\text{d}\:\text{s}\text{i}\text{z}\text{e}\times\:\:100}$$

On day six, animals were anesthetized with isoflurane, and the euthanasia of rats was performed by cervical dislocation (CD) according to the American Veterinary Medical Association (AVMA) Guidelines for the Euthanasia of Animals (2020 Edition). Hematoxylin-eosin (H&E) (Sigma, USA) staining was employed to analyze the skin lesions and collect samples of the afflicted area that ranged in thickness from 2 to 5 mm for histological investigation [36, 38].

### Statistical analysis

Data were analyzed using one-way analysis of variance (ANOVA), a T-test, and a p-value of 0.05 as the cut-off for significance. Each experiment was carried out three times, and the mean SD was used to express the findings.

## Results

### Bacterial isolates

Regarding the sources of clinical samples, most isolates were recovered from sputum (*n* = 78), followed by urine (*n* = 31), wounds (*n* = 7), and blood (*n* = 4). The isolates were maintained in TSB at -80 °C with 10% (v/v) glycerol for further studies.

### Antibiotic susceptibility testing of the recovered isolates

Concerning the collected *S. aureus* (*n* = 120) isolates, the result revealed that there were 70 MDR isolates (58.3%). The MDR isolates were 34 MRSA isolates (28.3 %). Most of the isolates were resistant to azithromycin (*n* = 101 strains, 84.1%). While all isolates were sensitive to vancomycin (See supplementary table TS1).

### Minimum inhibitory concentrations (MIC) of MDR isolates

According to CLSI (2018), the MICs of oxacillin were determined against MDR isolates (*n* = 70). The obtained result revealed that there was a wide range of MIC (0.5–256 µg/ml), as shown in Table 2.

**Table 2:** Minimum inhibitory concentration values of oxacillin for multi-drug resistance isolates

Oxacillin MIC (µg/ml)	Number of isolates	Isolate code
**0.5**	**2**	S 19, S 34.
**1**	**11**	S 8, S 10, S 22, S 29, S 31, S 32, S 75, S 77, W 4, U 94, U 99
**2**	**23**	S 13, S 21, S 24, S 35, S 37, S 65, S 68, S 69, S 72, S 70, S 79, S 80, W 1, W 2, U 90, U 91, U 101, U 103, U 104, U 106, U 112, U 113, U 118.
**4**	**4**	W 3, W 6, U 96, U 108.
**8**	**13**	S 14, S 15, S 20, S 23, S 36, S 45, S 46, S 51, S 63, S 66, U 92, U 115, B 88.
**16**	**3**	S 47, W 7, U 109.
**32**	**2**	S 58, U 119.
**64**	**8**	S 11, S 33, S 39, S 71, S 73, S 74, U 110, B 89.
**128**	**1**	W 5
**256**	**3**	S 78, U 93, B 86.

S; sputum, U; urine, W; wound, B; blood

### Biofilm production by MDR isolates

The ability of MDR isolates (*n* = 70) to produce biofilms was detected using a microtiter plate technique and optical density (OD) was determined. The result revealed the different capacities of biofilm formation by different bacterial isolates. Most isolates were moderate producers (*n* = 38,  54.3%) followed by strong (*n* = 21, 30%) and weak (*n* = 11,15.7%) as shown in Table 3.

**Table 3:** Biofilm formation by MDR isolates

Biofilm production	Percentage MDR isolates	Isolate code
**Strong**	**30%**	S 11, S 19, S 31, S 33, S 35, S 39, S 70, S 71, S 73, S 74, S 77, S 80, W 3, W 5, U 93, U96, U 99, U 109, U 110, B 86, B 89.
**Moderate**	**54.3%**	S 8, S 10, S 14, S 15, S 21, S 22, S 23, S 24, S 29, S 32, S 34, S 36, S 37, S 45, S 47, S 51, S 63, S 65, S 66, S 68, S 69, S 72, S 78, W 1, W 2, W 4, W 7, U 90, U 91, U 92, U 101, U 103, U 106, U 108, U 112, U 118, U 119, B 88.
**Weak**	**15.7%**	S 13, S 20, S 46, S 58, S 75, S 79, W 6, U 94, U 104, U 113, U 115.

S; sputum, U; urine, W; wound, B; blood

### Virulence factors production by selected MDR isolates

The virulence factors’ production by selected MDR isolates (*n* = 13) is presented in Table 4. The selected isolates were 10 MRSA and 3 non-MRSA isolates. There were 8 SBF and 5 MBF. All of them produce hemolysin and protease.

**Table 4:** Virulence factors production by selected MDR isolates (*n* = 13)

Isolate code	MRSA	Non- MRSA	Protease production	Hemolysin production	Biofilm production	
					Strong	Moderate
S 11	✓		✓	✓	✓	
S 14	✓		✓	✓		✓
S 33	✓		✓	✓	✓	
S 39	✓		✓	✓	✓	
S 51	✓		✓	✓		✓
S 71	✓		✓	✓	✓	
S 74	✓		✓	✓	✓	
W 7	✓		✓	✓		✓
U 110	✓		✓	✓		✓
B 89	✓		✓	✓	✓	
S 8		✓	✓	✓		✓
S 69		✓	✓	✓	✓	
S 80		✓	✓	✓	✓	

### Detection of *tst* gene in selected MDR isolates using PCR technique

**Fig. 1:**
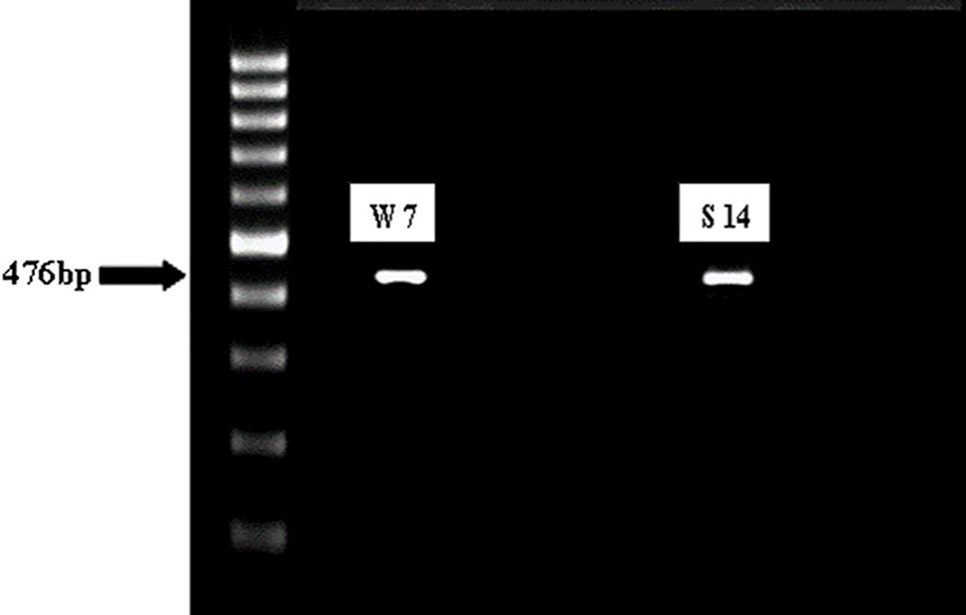
Agarose gel electrophoresis of DNA extract from 10 selected isolates showed that TSST1 gene with molecular weight 476 bp was detected in isolates 7 W and S 14

The selected isolates (*n* = 13) were screened for the presence of *tst* gene using the PCR technique. Two isolates showed the presence of a gene band with an amplicon size of 476 bp (Fig. 1).

### Bacterial growth in the presence of sub-MIC of oxacillin

**Fig. 2:**
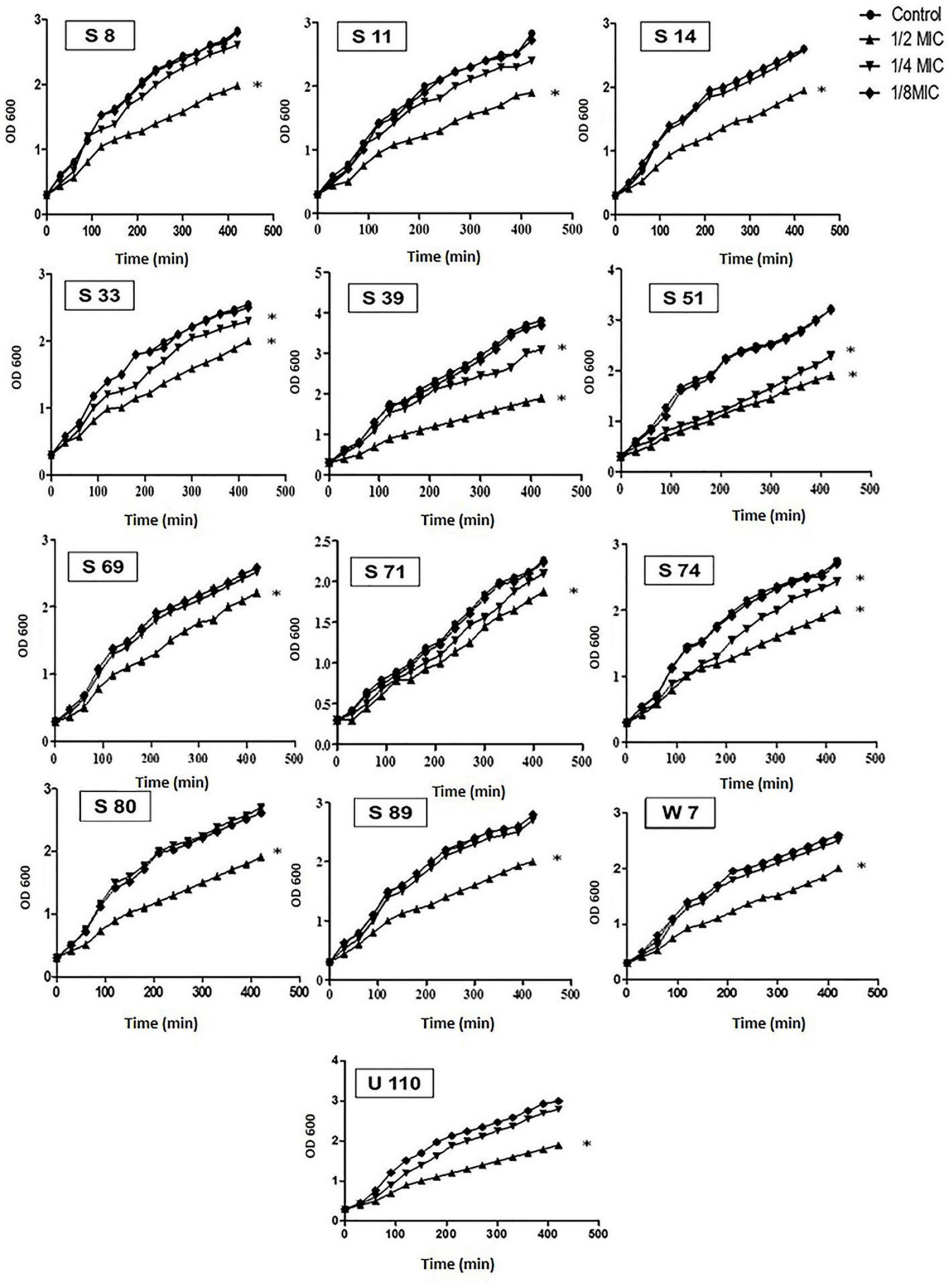
Growth curves of selected *S. aureus* isolates grown in the absence and presence of 1/2, 1/4, and 1/8 MICs of OX at different intervals of time. The code of each isolate is indicated on the graph. The asterisks represent statistical significance (*P* < 0.05)

The effect of OX at 1/2, 1/4, and 1/8 MICs on the bacterial growth of selected isolates (*n* = 13) was tested at different interval times. The obtained data showed that 1/2 markedly affects bacterial growth, while 1/4 MIC showed lower activity on bacterial growth, and the effect of 1/8 MIC was negligible (Fig. 2).

### Biofilm production in the presence of sub-MICs of oxacillin

#### Crystal Violet microtitration assay

**Fig. 3:**
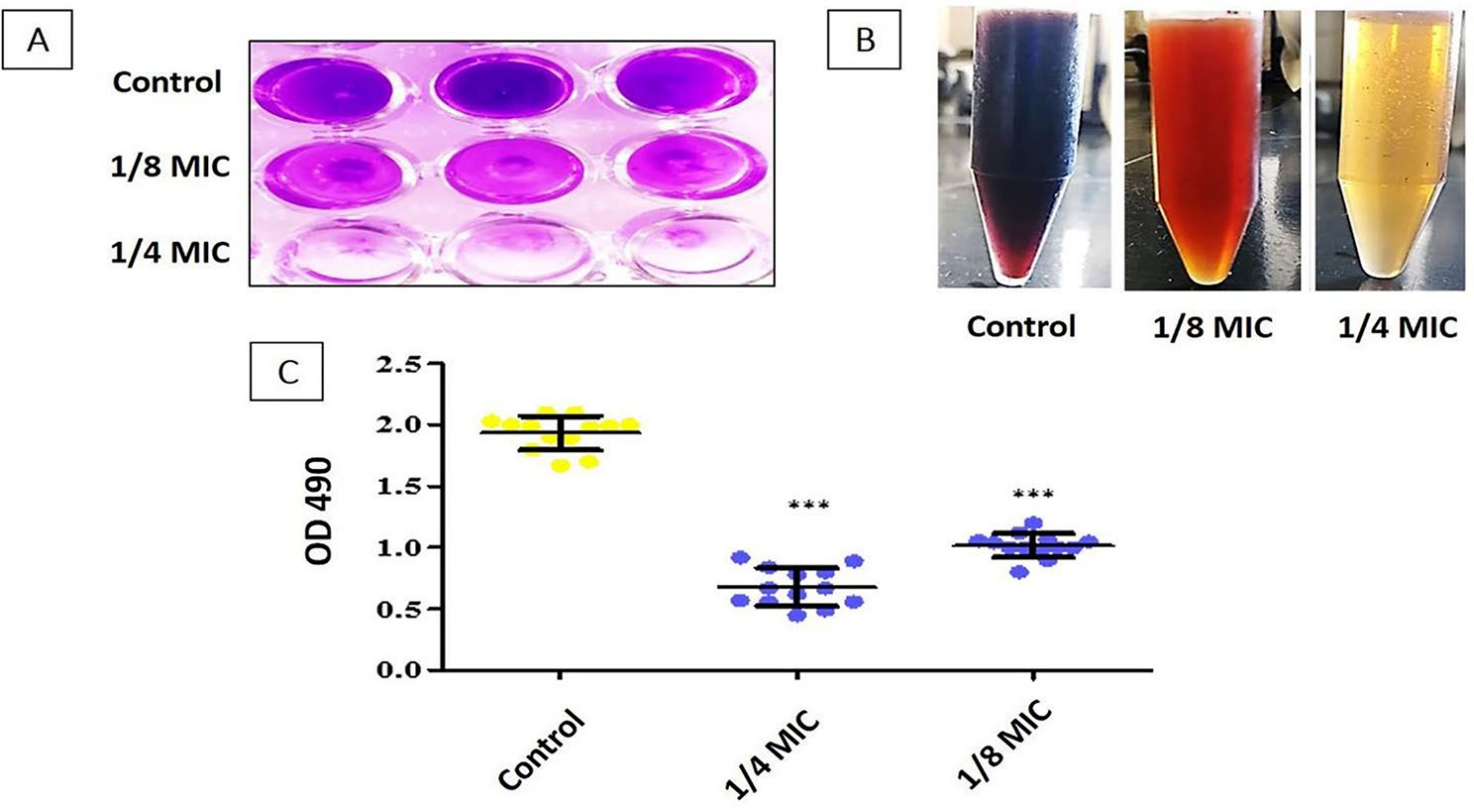
A representative review of the effect of OX sub-MIC on biofilm formation by (**A**) Crystal violet microtitration formation. **(B)** Phenol-sulfuric acid method to detect the alteration of EPS **(C)** Chart which revealed the change in OD values. The asterisks represent statistical significance (*P* < 0.05)

The biofilm creation of the selected isolates (*n* = 13) significantly decreased (*P* < 0.05) in the presence of OX as shown in Fig. 3A. The range of percentage of reduction was (8–40), and (18–64) after exposure to the 1/8 MIC and 1/4 MIC of OX, respectively (Table 5).

**Table 5:** The percentage of reduction of the bacterial biofilm (*n* = 13) in the presence of sub-MICs of oxacillin

Isolate code	S 11	S 14	S 33	S 39	S 51	S 71	S 74	W 7	U 110	B 89	S 8	S 69	S 80
**1/8 MIC**	23	8	28	16	20	40	24	28.5	26	26	13	22	25
**1/4 MIC**	37	18	64	21	23	43	49	31	31	31	18	37	36

#### Phenol-sulfuric acid method for assessment of EPS formation

The oxacillin at 1/4 and 1/8 MICs significantly decreases (*P* < 0.05) EPS formation which was detected by the decrease in the intensity of the red color as shown in Fig. 3B and C.

#### Confocal laser scanning microscopy (CLSM)

**Fig. 4:**
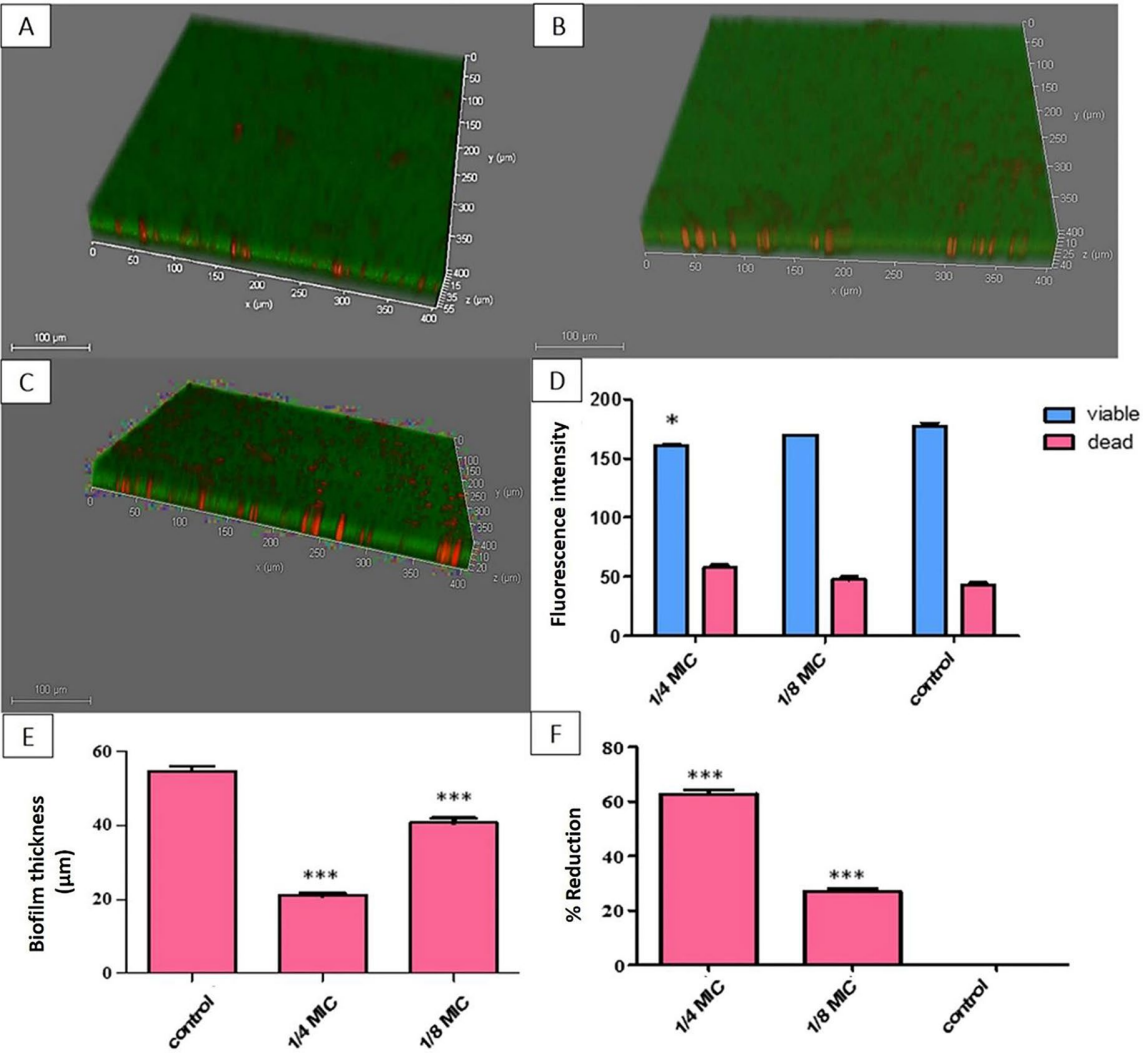
CLSM for S 33 isolate to detect biofilm thickness before and after treatment. (**A**) Untreated *S. aureus* biofilm. (**B**) Oxacillin-treated biofilm at 1/8 MIC. **(C)** Oxacillin-treated biofilm at 1/4 MIC. (**D**) Change fluorescence intensity with change viability with oxacillin treatment. **(E)** Biofilm thickness before and after oxacillin treatment. (**F**) The percentage of reduction in biofilm thickness after oxacillin treatment. The error bars indicate standard deviations. The asterisks represent statistical significance (*P* < 0.05)

The impact of 1/4 and 1/8 MICs of OX on *S. aureus* biofilm formation was confirmed using CLSM by the double-stained technique. The PI staining of the dead bacteria dyed them red, whereas the AO staining of the living bacteria stained them green. The percentage of reduction after exposure to 1/8 and 1/4 MICs was 27.3 and 63.3%, respectively (Fig. 4).

### Decrease *S. aureus* virulence with sub-MIC of oxacillin

**Fig. 5:**
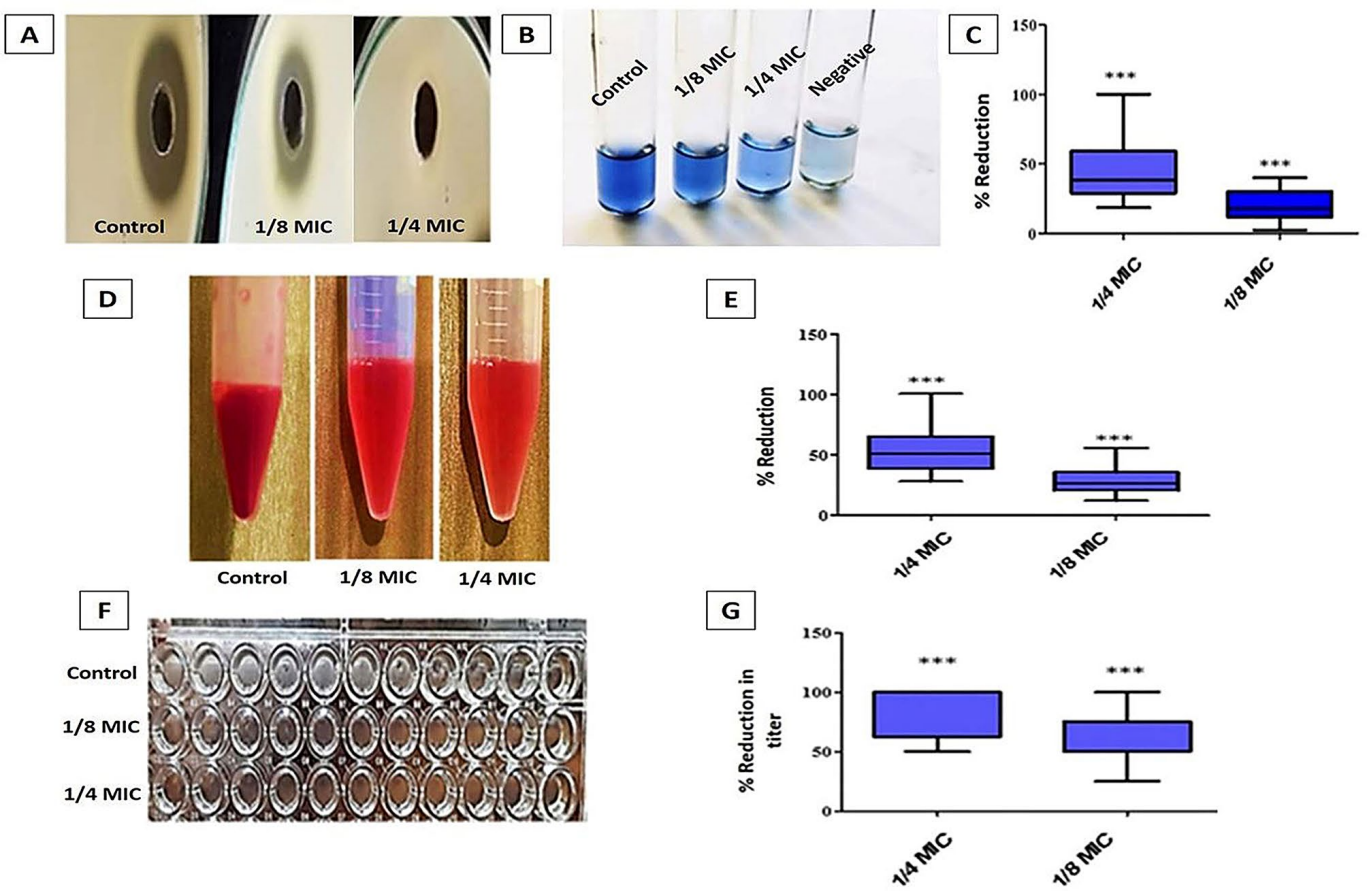
A representative result about the effect of OX at sub-MIC on *S.*aureus virulence factors’ production. (**A**) Skimmed milk agar for determination of protease production before and after 1/4 and 1/8 MIC of OX treatment. **(B)** Protease assay before and after OX treatment. **(C)** Percentage of reduction of protease production with different concentrations of OX. **(D)** Hemolytic activity of *S. aureus.***(E)** Percentage of reduction in hemolysin production with different concentrations of OX. **(F)** Coagulase quantification by determination of titer before and after treatment using a microtiter plate. **(G)** Percentage of the reduction in titer of coagulase after OX treatment. The error bars indicate standard deviations. The asterisks represent statistical significance (*P* < 0.05)

The results of oxacillin treatment at sub-MIC levels on the production of protease, hemolysis, coagulase, and TSST-1 were evaluated. The treated *S. aureus* showed a significant decrease (*P* < 0.05) in protease production by skimmed milk and protease assay methods, as shown in Fig. 5A and B. The proteolytic activity of *S. aureus* was inhibited by 18.7–100% and 2.3–40% for 1/4 MIC and 1/8 MIC of OX, respectively, as shown in Fig. 5C. The hemolytic activity of *S. aureus* isolates was detected with and without 1/4 and 1/8 MICs of OX, and the degree of hemolysis was determined using the spectrophotometric method. Oxacillin significantly decreased the hemolytic activity of *S. aureus* isolates, as shown in Fig. 5D. The hemolytic activity was significantly (*P* < 0.05) decreased by (28–100%) and (12–56%) for 1/4 and 1/8 MICs of OX, respectively (Fig. 5E). Concerning coagulation titer, the result showed that OX at 1/4 and 1/8 MICs significantly decreased coagulase production, as shown in Fig. 5F. Coagulase production was significantly (*P* < 0.05) decreased after treatment with 1/4 and 1/8 MICs of OX, as shown in Fig. 5G.

### Morphological changes induced by sub-MIC of oxacillin

#### Scanning electron microscope (SEM)

**Fig. 6:**
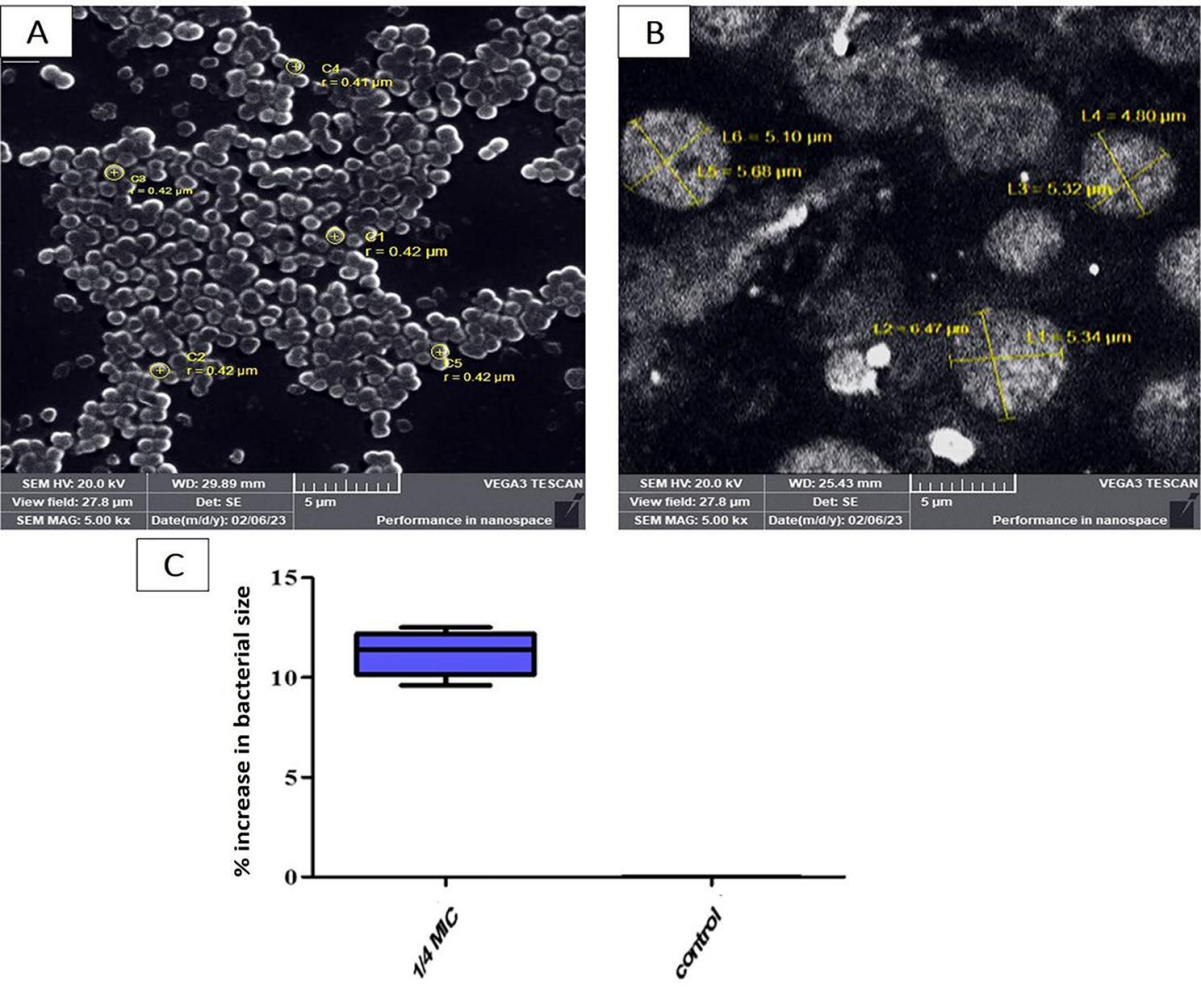
SEM showing the effect of sub-MIC of OX on bacterial morphology for S 33 isolate. (**A**) Untreated *S. aureus* reveals a spherical cell. **(B)** Treated *S. aureus* with 1/4 MIC of OX cell showing enlargement and elongation in the bacterial cell **(C)** Percentage increase in bacterial size with treatment

Exposing *S. aureus* to sub-MIC OX causes cell enlargement, breakdown,, and/or developing cells with holes. The results revealed that after exposing *S. aureus* to 1/4 MIC of OX, there was an enlargement in the cell ranging from (9.5–12.5 μm) with a degree of elongation in the cell, while the untreated cell showed a spherical shape without any elongation in its shape (Fig. 6).

#### Transmission electron microscope (TEM)

**Fig. 7:**
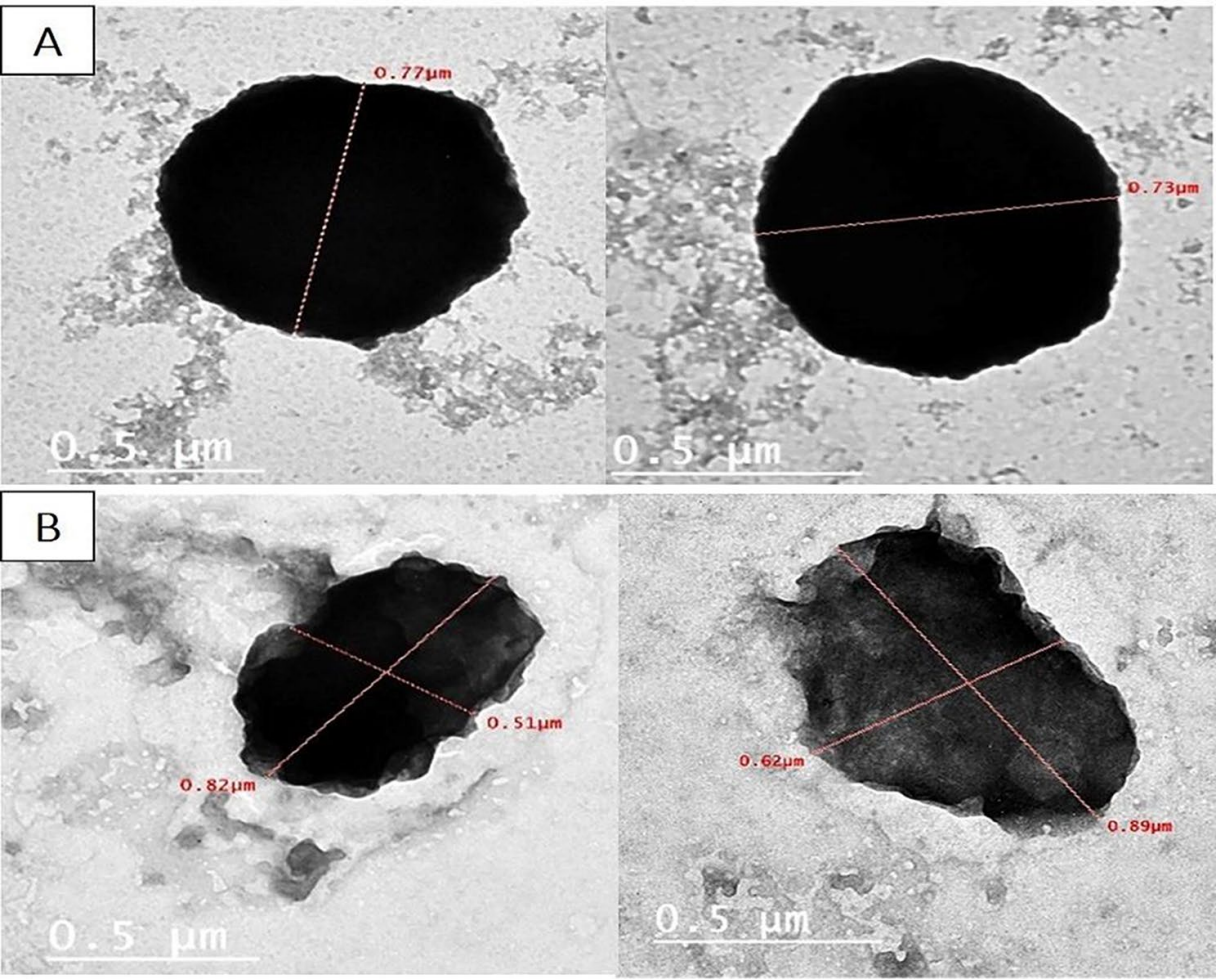
TEM shows the change in bacterial morphology with sub-MIC of OX treatment for S 33 isolate. (**A**) *S. aureus* (control) strain showing spherical shape and intact cell wall and cell membrane. **(B)** Treated bacterial isolate with 1/8 MIC of OX shows elongation with changes in the cell wall and cell membrane

Data revealed that there is elongation in the bacterial isolate and an increase in bacterial size with changes in the cell wall and cell membrane integrity (Fig. 7A and B).

#### TEM with ultra-thin section formation

**Fig. 8:**
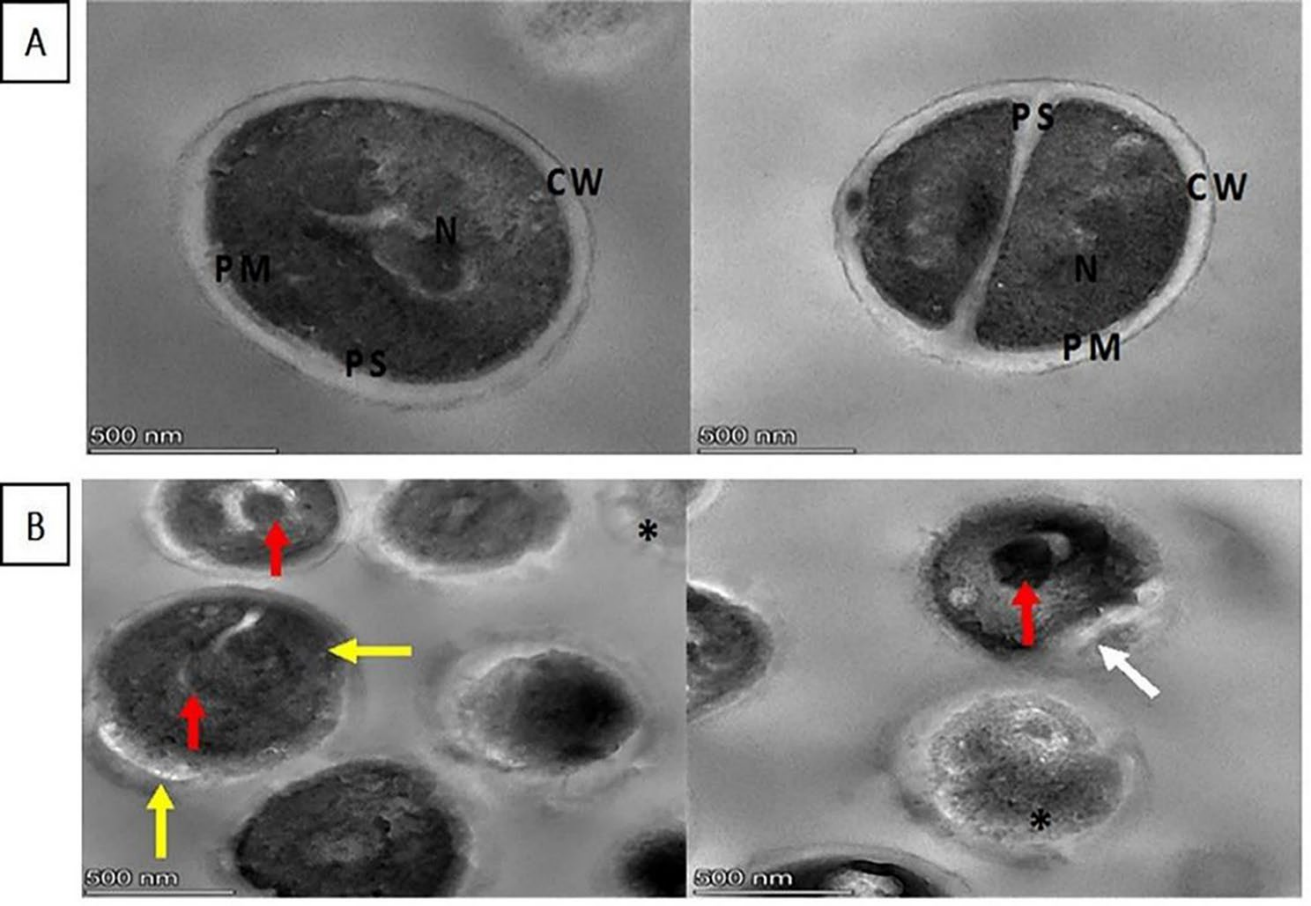
TEM showing the change in bacterial morphology with 1/8 MIC of OX treatment for S 33 isolate after making an ultra-thin section in the bacterial pellet. (**A**) Normal cells showing the typical compartments and structures are round and intact, with a well-defined plasma membrane (PM), periplasmic space (PS), and cell wall (CW). The intracellular DNA region exhibits a highly homogeneous electron density as a nucleoid (N). **(B**) Treated cells showing some mesosome-like structures and non-membrane-enclosed bodies (red arrow), some completely lysed cells and necrotic (*), the cell wall with different thicknesses (yellow arrow), and invagination of periplasmic space (white arrow)

Our result revealed the changes that occur in the bacterial morphology after exposure to 1/8 MIC of OX, showing that some mesosome-like structures are formed and non-membrane-enclosed bodies are formed, some cells are completely lysed and necrotic, the cell wall has different thicknesses and invaginations of periplasmic space occur (Fig. 8A and B).

### The effect of sub-MIC of oxacillin on gene expression using qRT-PCR

**Fig. 9:**
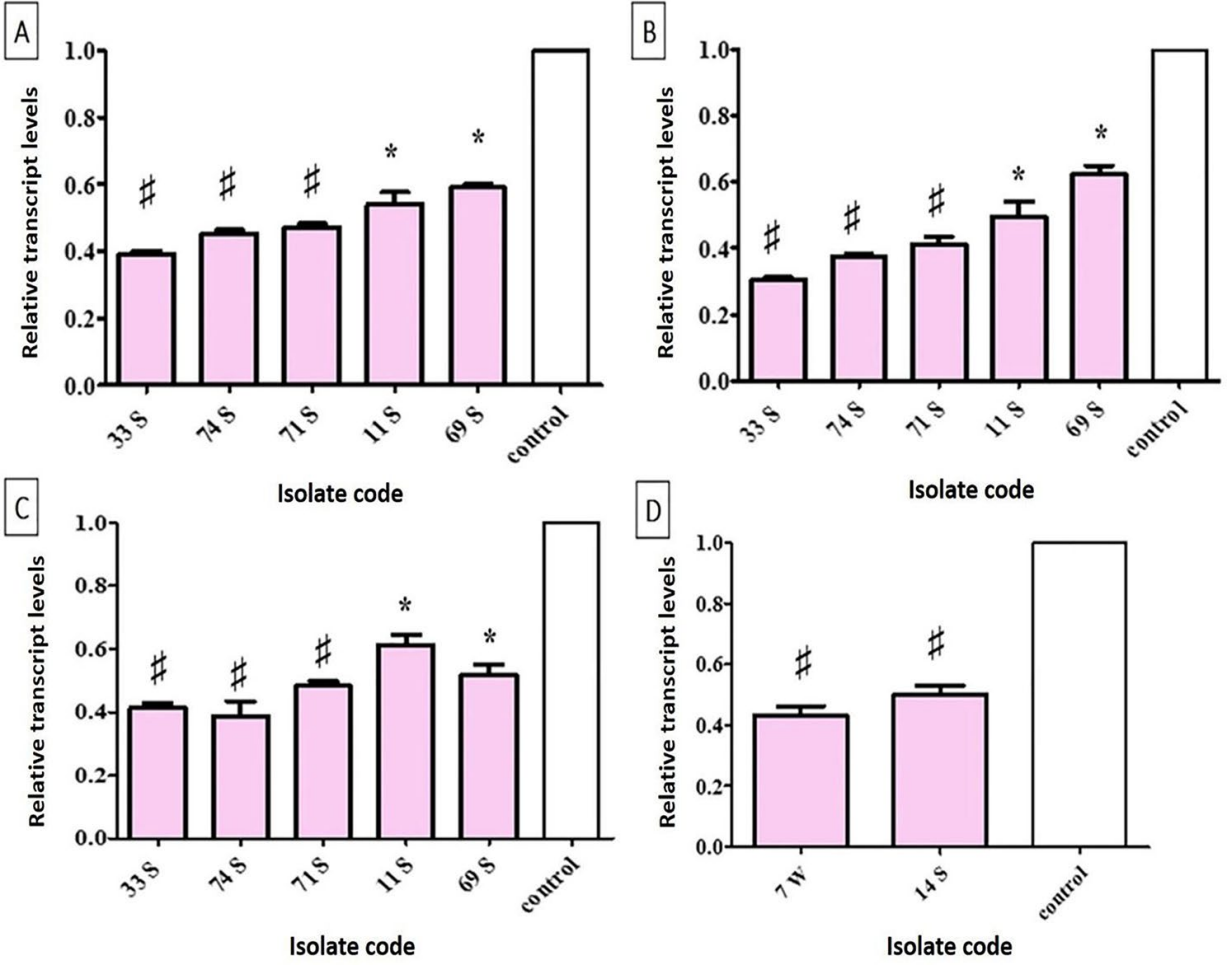
Relative transcript levels after 1/4 MIC of OX treatment by using qRT-PCR (**A**) Relative transcript levels in the *agrA* gene after oxacillin treatment. (**B**) Relative transcript levels in the *icaA* gene after oxacillin treatment. (**C**) Relative transcript levels in the *coa* gene after oxacillin treatment. (**D**) Relative transcript levels in the *tst* gene after oxacillin treatment show that there was a decrease in the gene expression after exposure to 1/4 MIC of OX, the error bars indicate standard deviations. ♯ means the change in the gene expression is two-fold or more. The asterisks mean the change in gene expression is more than one-fold but less than two-fold

The exact mechanism by which sub-MIC of OX affected *S. aureus* isolate biofilm and virulence factors’ production (*n* = 5) was investigated using qRT-PCR by studying the effect on biofilm and other virulence-associated genes such as *agrA*, *icaA*, *coa*, *and tst* genes. The *agrA* gene expression diminished following OX treatment in each studied isolate, and the reduced percentages in the *agrA* gene expression levels were 61, 54, 52, 45, and 42% for *S. aureus* isolates S 33, S 74, S 71, S 11, and S 69, respectively (Fig. 9A). The *icaA* gene expression diminished following OX treatment in each studied isolate, and the reduced percentages in the *icaA* gene expression levels were 70, 62.2, 58, 46, and 40% for *S. aureus* isolates S 33, S 74, S 71, S 11, and S 69, respectively (Fig. 9B). The *coa* gene expression diminished following OX treatment in each studied isolate, and the reduced percentages in the *coa* gene expression levels were 57, 56.2, 35, 45, and 40% for *S. aureus* isolates S 33, S 74, S 71, S 11, and S 69, respectively (Fig. 9C). The *tst* gene expression diminished following OX treatment in the 2 isolates that PCR revealed to carry the gene, and the reduced percentages in the *tst* gene expression levels were 55 and 47% for *S. aureus* isolates S 7 and S 14, respectively (Fig. 9D).

### In vivo assay using wound infection model

**Fig. 10:**
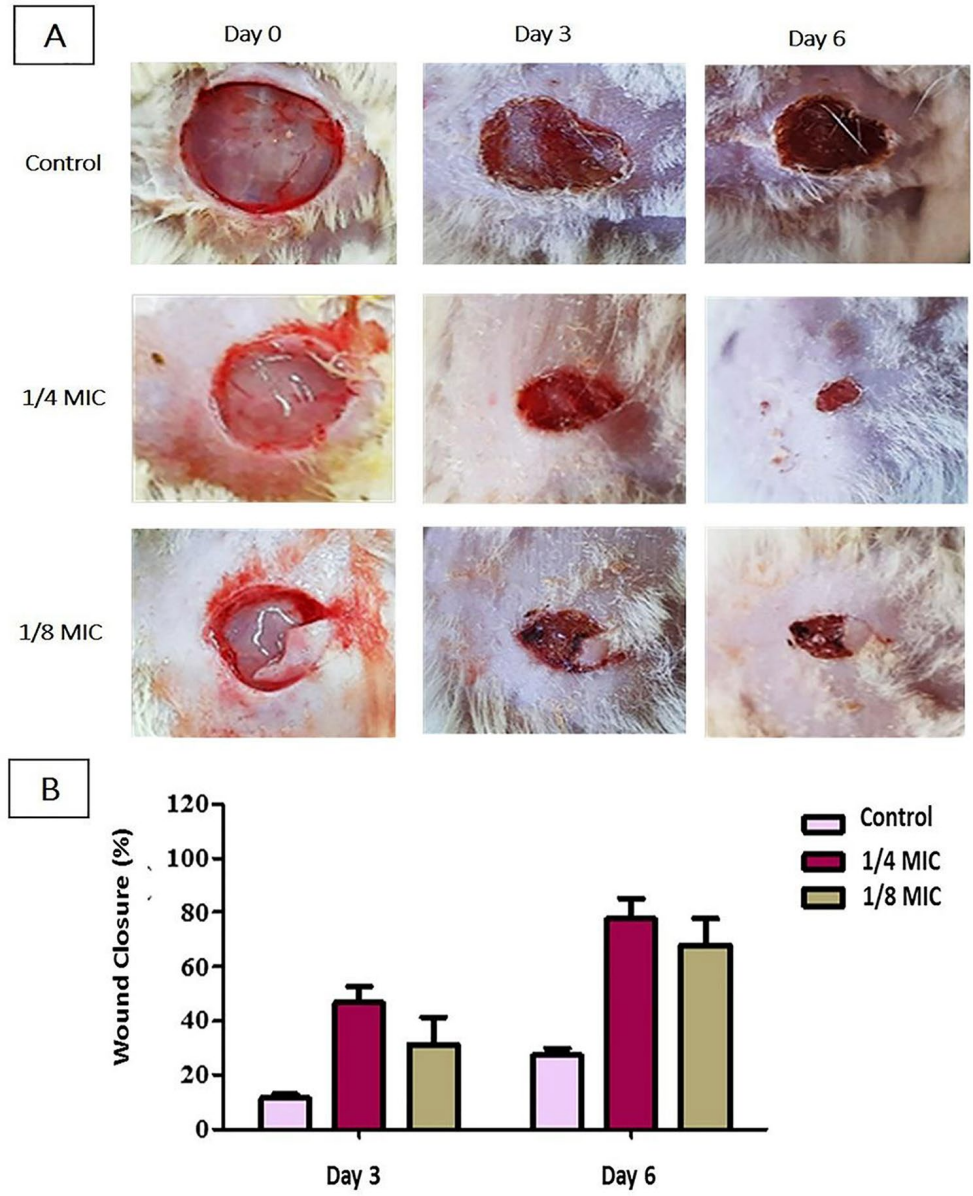
The effect of sub-MIC of oxacillin on controlling *S. aureus* skin wound infection. (**A**) Photos of representative rats of each group (treated with vehicle vs. oxacillin). (**B**) Percentage of wound closure before and after treatment

The wound closure rates were found to be time-dependent in both the treated and untreated groups, with the treated rats’ wound closure being significantly (*P* > 0.05) higher than the untreated rats (Fig. 10A and B).

### Histopathological examination of skin wound

**Fig. 11:**
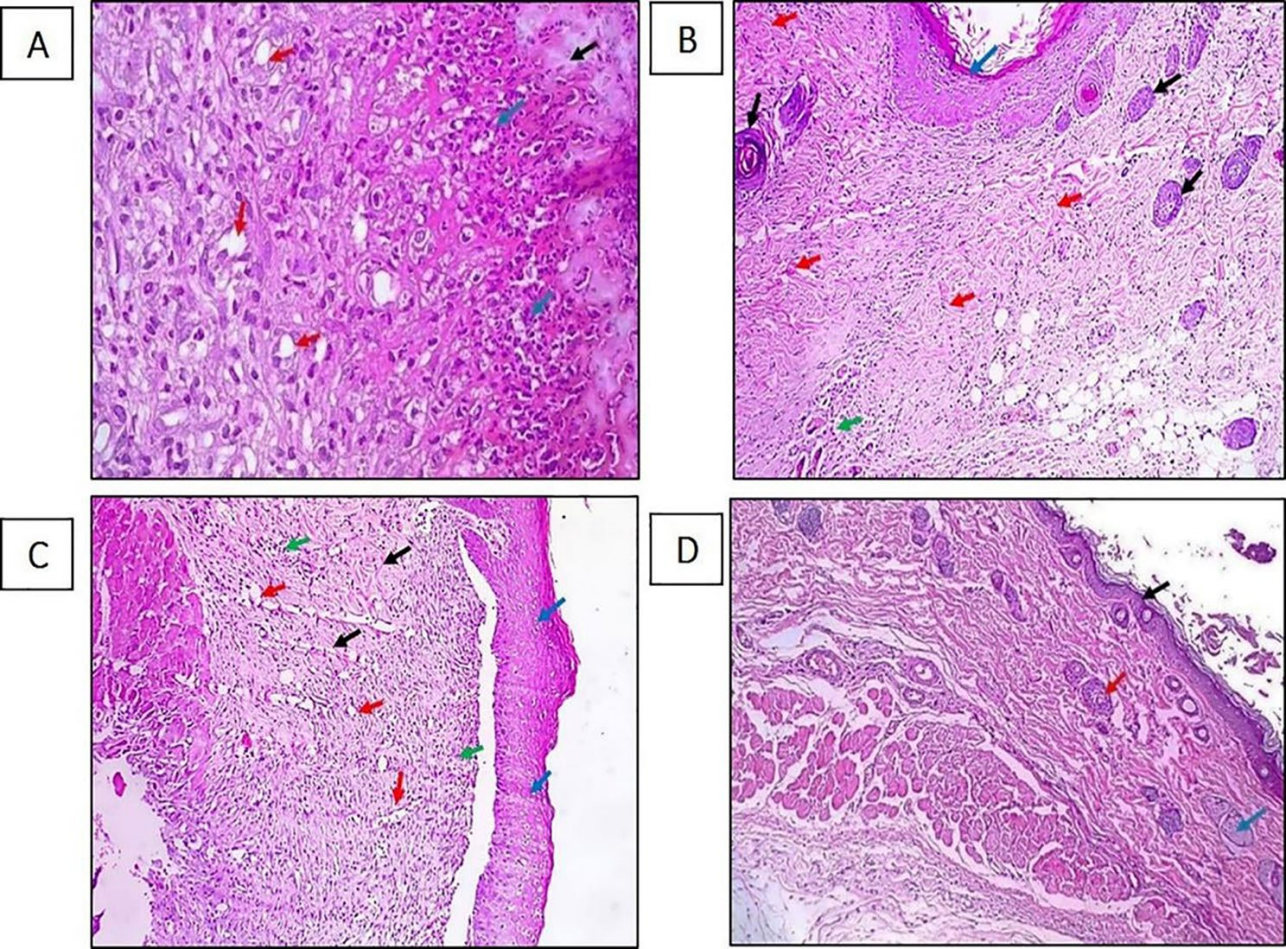
Representative images of H and E histological sections of the skin tissue of male rats sacrificed after 6 days. **(A) Group I** (positive control): a section in the skin showed a scab (black arrow) with heavy inflammation [acute and chronic inflammatory cells] (blue arrows) with newly formed blood vessels (red arrows). **(B) Group II** (treated with 1/4 MIC of OX) sections in the skin showed extensive epidermal regeneration (blue arrow) with underlying slight inflammation (green arrow) surrounded by extensive activated hair follicles (black arrows), extensive collagen deposition (red arrows). **(C) Group III** (treated with 1/8 MIC of OX): section in skin showed moderate epidermal regeneration (blue arrows) with underlying moderate inflammation (green arrows) surrounded by moderate collagen deposition (black arrows) and extensive granulation tissue formation with moderate angiogenesis (red arrows). **(D) Negative control** showed normal skin with intact keratinized epidermis (black arrow) with underlying dermis showing hair follicles (red arrow) and sebaceous glands (blue arrow) with no inflammation

The ability of oxacillin in different concentrations to improve wound healing was evaluated using H&E stains. The histological analysis of tissues in the *S. aureus*-infected and untreated group (Group I) showed scabs (black arrows) with heavy inflammation [acute and chronic inflammatory cells] with newly formed blood vessels (Fig. 11A). Furthermore, the histopathological analysis of the *S. aureus* infected and treated with 1/4 MIC of OX (Group II) revealed extensive epidermal regeneration with underlying slight inflammation surrounded by extensive activated hair follicles and extensive collagen deposition, as shown in Fig. 11B. *S. aureus* infected, and the group treated with 1/8 MIC of OX (Group III) showed moderate epidermal regeneration with underlying moderate inflammation surrounded by moderate collagen deposition and extensive granulation tissue formation with moderate angiogenesis (Fig. 11C). The negative control showed normal skin with intact keratinized epidermis (black arrow) with underlying dermis showing hair follicles (red arrow) and sebaceous glands (blue arrow) with no inflammation (Fig. 11D).

### Scoring of wounds

Scoring of treated and untreated wounds showed that there was an increase in wound angiogenesis and proliferation of fibroblasts, collagen deposition-activated hair follicles, epidermal regeneration, and a decrease in inflammatory cell infiltrate and scab formation with sub-MIC treatment, which support wound healing (Table 6).

**Table 6:** Scoring histological parameters of oxacillin-treated and untreated wounds

Histological parameters	Parameter scoring of		
	Untreated	Oxacillin treated at	
	Positive control	1/8 MIC	1/4 MIC
**Epidermal regeneration**	**-**	**++**	**+++**
**Granulation tissue**	**++**	**++**	**+++**
**Inflammatory cells infiltrate**	**+++**	**++**	**+**
**Scab formation**	**+++**	**+**	**-**
**Angiogenesis**	**++**	**++**	**+++**
**Proliferation of fibroblasts**	**-**	**++**	**+++**
**Collagen deposition**	**-**	**++**	**+++**
**Activated hair follicles**	**-**	**++**	**+++**

+ means slight, ++ means moderate, +++ means Extensive, and – means absent

## Discussion

*Staphylococcus aureus* is a serious human pathogen with an increasing risk to the public’s health. Serious skin infections, necrotizing pneumonia, and bacteremia are only a few of the illnesses that *S. aureus* can cause [39]. The primary factor causing *S. aureus* widespread distribution and drug resistance is the ability to form a biofilm, which can be found on both biotic and abiotic surfaces [40]. The polysaccharide intercellular adhesin (PIA) is a crucial element of *S. aureus* biofilms and is produced by the intercellular adhesion (ica) locus. Biofilm formation is also linked to *ica* gene expression [41, 42]. In our trial to find a drug that may be used to combat the increasing resistance of *S. aureus*, we investigated the effect of oxacillin in the modified concentration on the isolate’s ability to form biofilm.

Our results revealed that biofilm development is considerably reduced by sub-MIC (1/4 and 1/8) of OX by approximately 64% and 40%, respectively, using a crystal violet assay in addition to a phenol-sulfuric acid method, which revealed a reduction in biofilm matrix (EPS) formation detected by the decrease in the color intensity. Further investigation of the biofilm performed using CLSM revealed the significance of biofilm reduction 27% and 63.3% after 1/8 and 1/4 MIC of OX treatment, respectively. The previous results were confirmed using QRT-PCR, which in turn revealed a reduction in *ica A* gene expression by 46–70%.

Previous studies also showed a similar result to ours; Kaplan et al. and Stoitsova et al. showed that the pattern by which beta-lactam affected the biofilm is not only influenced by the antibiotic and concentration but also strain-dependent [43, 44]. Moreover, Frank et al. mentioned that cell wall-active antibiotics at sub-MIC levels either had no impact or had an inhibitory effect on *S. aureus* biofilm development [45]. In the Mjidpour study, it was reported that *S. aureus* treated with OX at sub-MIC levels significantly reduced the production of biofilms, except for one isolate that showed induction in biofilm [46]. Furthermore, beta-lactams have anti-biofilm activity against *Streptococcus pyogenes*, according to Šmitran study [47]. For that, the anti-biofilm exerted by sub-MIC OX treatment can be used to combat the *S. aureus* resistance and attenuate the bacterial infection severity. Moreover, the virulence factors that *S. aureus* produces influence how harmful the infection will be, such as adhesins, secreted enzymes, and toxins [7, 48–50].

Through our research, we also studied the impacts of OX on *S. aureus* virulence factor production. It was observed that there is a significant reduction in protease by 18.7–100% and 2.3–40% after 1/4 and 1/8 MIC treatment, respectively. Hemolysin production was significantly reduced by 28–100% and 12–56% after 1/4 and 1/8 MIC treatment, respectively. Furthermore, coagulase production was significantly decreased after OX treatment, approximately up to 100%. The exact mechanism by which virulence factors were inhibited was determined by performing QRT-PCR, and the ranges of reduction in *agrA*,* coa*, and *tst* gene expression were 42–61%, 35–57%, and 47–55%, respectively.

In agreement with our study, El-Mowafy et al. revealed that low doses of beta-lactam decreased virulence factors and significantly affected QS signals [51]. Additionally, Kumar et al. and Viedma et al. documented that some beta-lactams have anti-QS and anti-virulence activities [52, 53]. Furthermore, Asadi and Derakhshan reported that, compared to untreated isolates, isolates treated with a sub-MIC of beta-lactam antibiotics exhibited lower levels of hemolysis [54, 55]. Moreover, Haddadin et al. proved that exposure to sub-MIC beta-lactam decreases TSST-1 production [33]. Additionally, Tibúrcio et al. reported that sub-MICs of beta-lactam reduce biofilm development and virulence factor production by interfering with the gene’s expression [56].

Studying the effect of OX at sub-MIC on bacterial morphology using SEM and TEM illustrated the changes that occurred in the cell wall, cell membrane integrity, and pore formation, as well as the elongation and enlargement of the cell. The morphological changes that occurred after exposure to sub-MIC are strain-dependent and antibiotic-dependent. This was also reported by Chen et al., who classified the morphological changes that occur after exposure to sub-MIC OX into three different forms of morphological alterations, including cell wall collapse, cell wall component modifications, and cell morphology deformation [6]. Also, Zhanel et al. investigated the implications of sub-MIC beta-lactam, and the result revealed that the effect is antibiotic-dependent and strain-dependent, but mainly sub-MIC beta-lactam produces excessively big cells with thickened cross-walls with minor changes to the outer cell walls [57].

Finally, we examined the effect of OX at 1/4 and 1/8 MIC on wound healing in rats, and the result showed that OX increased wound healing. This was concluded from the calculation of % of wound closure and confirmed by histopathological examination, which further indicates the increase in epidermal regeneration, collagen deposition, granulation tissue formation, and hair follicles with the decrease in inflammation. This was also reported by Zhanel et al., who revealed that rabbits given beta-lactams at subinhibitory concentrations showed altered bacterial morphology and had longer survival rates [57]. Moreover, this protective effect was also detected by Nolan & Behrends’s study, which illustrated that sub-MIC exposure to ceftazidime, tetracycline, ciprofloxacin, and tobramycin shields rats against *P. aeruginosa-*induced lung infections [58].

From the phenotypic and genotypic studies, we revealed that the sub-MIC of OX not only affected biofilm and virulence factors outwardly but also affected gene expression, which was confirmed by a rat wound healing experiment that revealed significant wound healing, a reduction in inflammation, and scab formation after treatment with sub-MICs of OX. This can open the door for using this approach to control *S. aureus* resistance and decrease patient outcomes.

## Conclusion

From our study, it was detected that there is an increase in bacterial resistance. This in turn leads to a worsening of the patient’s outcome and prolongs the required period for a complete recovery. The bacterial ability to form biofilm and secrete different virulence factors is a very critical point for establishing the infection and reducing the bacterial sensitivity, so there is a great interest in finding new drugs instead of traditional ones to combat the bacterial resistance and decrease the infection severity. The reuse of the existing antibiotic at its 1/4 or 1/8 MICs is very useful as it was found that it caused a significant reduction in both biofilm and virulence factor production and gives the body a chance to fight the infection without needing further investigation for its safety and side effects as it has already been used for decades. So, from our point of view, it is very important to reconsider the use of oxacillin in combating and attenuating *S. aureus* infection; however, further studies should be conducted to confirm these results. Until then, OX should still be used at an optimally high dose.

### Electronic supplementary material

Below is the link to the electronic supplementary material.

Supplementary Material 1

## Data Availability

All data generated or analyzed during this study are included in this article.
