# Supplementary material for: Potential antivirulence and antibiofilm activities of sub-MIC of oxacillin against MDR S. aureus isolates: an in-vitro and in-vivo study
Source: BMC Microbiol. 2024 Aug 9;24:295. doi: 10.1186/s12866-024-03429-8 (PMC11312681; doi:10.1186/s12866-024-03429-8)
Supplement: Supplementary file 1 — Supplementary Material 1 [file 12866_2024_3429_MOESM1_ESM.docx]

**Table (TS1). Antimicrobial resistance pattern of the recovered 120 *S. aureus* isolates.**

(P) penicillin, (OX) oxacillin, (FOX) cefoxitin, (AZM) azithromycin, (C) chloramphenicol, (SXT) trimethoprim-sulfamethoxazole, (LZ) linezolid, (LEV) levofloxacin, (T) tetracycline, (CD) clindamycin, (RA) rifampicin, (V) vancomycin, (CIP) ciprofloxacin, (GN) gentamicin, (AK) amikacin.

| **Antimicrobial resistance patterns** | | **Pattern incidence** | **Isolates exhibiting the patterns** | **MAR index** |
| --- | --- | --- | --- | --- |
| **Pattern code** | **Resistance markers** |  |  |  |
| **I** |  |  |  |  |
| a | **AZ** | **9** | **S 30, S 49, S 56, S 59, S 60, S 61, S 29, S 81, U 107** | **0.06** |
| b | **P** | **4** | **S 57, S 62, S 84, U 95** | **0.06** |
| c | **C** | **2** | **S 81, S 82** | **0.06** |
| d | **SXT** | **1** | **U 100** | **0.06** |
| e | **CIP** | **1** | **U 102** | **0.06** |
| f | **LEV** | **4** | **U 116, U 117, S 67, S 42.** | **0.06** |
| **II** |  |  |  |  |
| a | **RA-CIP** | **1** | **U 120** | **0.13** |
| b | **P-AZM** | **18** | **U 114, U 97, U 98, S 85, S 83, S 55, S 52, S 53, S 43, S 48, S 44, S 38, S 40, S 41, S 25, S 26, S 9, S 17** | **0.13** |
| c | **RA-AZM** | **2** | **U 111, S 50** | **0.13** |
| d | **T-AZM** | **2** | **U 105, S 12** | **0.13** |
| e | **LZ-AZM** | **1** | **B 87** | **0.13** |
| f | **C-AZM** | **4** | **S 28, S 18, S 76, S 27** | **0.13** |
| g | **P-C** | **1** | **S 54** | **0.13** |
| h | **SXT-AZM** | **1** | **S 16** | **0.13** |
| **III** |  |  |  |  |
| a | **AZM-GEN-P** | **1** | **U 90** | **0.2** |
| b | **AZM-CIP-P** | **3** | **S 13, S 75, S 65** | **0.2** |
| c | **AZM-C-P** | **3** | **S 19, S 64** | **0.2** |
| **Antimicrobial resistance patterns** | | **Pattern incidence** | **Isolates exhibiting the patterns** | **MAR index** |
| **Pattern code** | **Resistance markers** |  |  |  |
| d | **AZM-CD-P** | **1** | **S 10** | **0.2** |
| e | **AZM-C-T** | **2** | **S 32, W 1** | **0.2** |
| f | **AZM-RA- P** | **2** | **S 21, W 4** | **0.2** |
| g | **AZM-LZ-P** | **1** | **S 8** | **0.2** |
| h | **FOX-OX-AZM** | **1** | **S 46** | **0.2** |
| i | **AZM-T-P** | **1** | **W 2** | **0.2** |
| **IV** |  |  |  |  |
| a | **AZM-T-SXT-GEN** | **1** | **U 91** | **0.267** |
| b | **AZM-SXT-GEN-P** | **1** | **S 77** | **0.267** |
| c | **AZM-C-T-SXT** | **1** | **U 94** | **0.267** |
| d | **AZM-C-T-P** | **1** | **S 24** | **0.267** |
| e | **AZM-P-CIP-LEV** | **4** | **U 101, U 103, U 113, S 35** | **0.267** |
| f | **AZM-T-GEN-CIP** | **1** | **U 104** | **0.267** |
| h | **AZM-T-CIP-LEV** | **1** | **U 112** | **0.267** |
| i | **AZM-C-P-CIP** | **1** | **S 68** | **0.267** |
| j | **AZM-C-P-LEV** | **2** | **S 69, S 70** | **0.267** |
| k | **AZM-T-FOX-OX** | **1** | **S 20** | **0.267** |
| l | **AZM-P-FOX-OX** | **2** | **B 89, U 115** | **0.267** |
| m | **C-P-FOX-OX** | **1** | **U 109** | **0.267** |
| n | **AZM-P-CIP-GEN** | **1** | **S 80** | **0.267** |
| o | **GEN-P-CIP-LEV** | **1** | **S 22** | **0.267** |
| P | **LZ-P-CIP-LEV** | **1** | **S 31** | **0.267** |
| **V** |  |  |  |  |
| **a** | **AZM-C-P-FOX-OX** | **3** | **W 7, S 47, S 51** | **0.33** |
| **Antimicrobial resistance patterns** | | **Pattern incidence** | **Isolates exhibiting the patterns** | **MAR index** |
| **Pattern code** | **Resistance markers** |  |  |  |
| b | **AZM-P-LZ-FOX-OX** | **1** | **S 66** | **0.33** |
| c | **AZM-T-P-FOX-OX** | **1** | **S 73** | **0.33** |
| d | **AZM-P-RA-FOX-OX** | **2** | **U 92, W 6** | **0.33** |
| e | **AZM-P-CIP-LEV-RA** | **1** | **S 34** | **0.33** |
| f | **AZM-P-CIP-LEV-SXT** | **1** | **S 37** | **0.33** |
| g | **AZM-C-P-CIP-LEV** | **1** | **S 72** | **0.33** |
| h | **AZM-T-P-CIP-LEV** | **1** | **U 106** | **0.33** |
| i | **AZM-GEN-P-CIP-SXT** | **1** | **U 118** | **0.33** |
| j | **AZM-P-CIP-LEV-GEN** | **1** | **S 79** | **0.33** |
| k | **AZM- CIP-P-FOX-OX** | **1** | **W 3** | **0.33** |
| l | **AZM-CIP-LEV-FOX-OX** | **1** | **U 96** | **0.33** |
| **VI** |  |  |  |  |
| a | **AZM-C-P- CD-FOX-OX** | **1** | **S 15** | **0.4** |
| b | **C-P-CIP-CD-FOX-OX** | **1** | **S 33** | **0.4** |
| c | **AZM-C-T-P-FOX-OX** | **2** | **S 39, S 74** | **0.4** |
| d | **C-P-CIP-LEV-FOX-OX** | **1** | **U 119** | **0.4** |
| e | **AZM-P-CIP-LEV-CD-RA** | **1** | **U 99** | **0.4** |
| f | **AZM-C-P-CIP-FOX-OX** | **1** | **S 71** | **0.4** |
| g | **AZM-CIP-LEV-RA-FOX-OX** | **1** | **U 108** | **0.4** |
| **Antimicrobial resistance patterns** | | **Pattern incidence** | **Isolates exhibiting the patterns** | **MAR index** |
| **Pattern code** | **Resistance markers** |  |  |  |
| **VII** |  |  |  |  |
| a | **AZM-C-T-P-CD-FOX-OX** | **3** | **S 23, S 45, S 36** | **0.46** |
| b | **AZM-T-P-LZ-CD-FOX-OX** | **1** | **S 63** | **0.46** |
| c | **C-SXT-P-CIP-LZ-FOX-OX** | **1** | **B 86** | **0.46** |
| **VIII** |  |  |  |  |
| a | **AZM-C-P-LEV-CD-RA-FOX-OX** | **1** | **S 14** | **0.53** |
| b | **AZM-P-CIP-LEV-CD-RA-FOX-OX** | **1** | **S 58** | **0.53** |
| c | **AZM-SXT-P-CIP-LEV-RA-FOX-OX** | **1** | **U 93** | **0.53** |
| **IX** |  |  |  |  |
| a | **AZM-GEN-CIP-LEV-P-LZ-T-FOX-OX** | **1** | **W 5** | **0.6** |
| b | **AZM-P-T-CIP-LEV-LZ-RA-FOX-OX** | **1** | **S 11** | **0.6** |
| c | **AZM-C-SXT-P-CIP-LEV-CD-FOX-OX** | **1** | **S 78** | **0.6** |
| **X** |  |  |  |  |
| a | **AZM-C-T-SXT-P-CIP-LEV-RA-FOX-OX** | **1** | **B 88** | **0.67** |
| **XI** |  |  |  |  |
| a | **AZM-C-T-SXT-P-CIP-LEV-LZ-RA-FOX-OX** | **1** | **U 110** | **0.73** |
